# EPB41L4A-AS1 and UNC5B-AS1 have diagnostic and prognostic significance in osteosarcoma

**DOI:** 10.1186/s13018-023-03754-0

**Published:** 2023-03-30

**Authors:** Ying Yan, Xiaochuan Liu, Yamei Li, Jingyi Yan, Ping Zhao, Lu Yang

**Affiliations:** 1 Shanghai Baoshan Center for Disease Control and Prevention, Shanghai, 201901 China; 2 Clinical Research Center, Shanghai Baoshan Luodian Hospital, No. 121 Luoxi Road, Baoshan District, Shanghai, 201908 China; 3 Juquan New Town Community Health Service Center, Gucun Town, Baoshan District, Shanghai, 201907 China

**Keywords:** Osteosarcoma, Prognosis, EPB41L4A-AS1, UNC5B-AS1

## Abstract

**Background:**

Deregulation of lncRNAs has been observed in human osteosarcoma. This study explored the diagnostic and prognostic significance of EPB41L4A-AS1 and UNC5B-AS1 in osteosarcoma.

**Methods:**

Relative levels of EPB41L4A-AS1 and UNC5B-AS1 were detected in osteosarcoma tissue samples and cells. The ability to distinguish osteosarcoma from health was assessed by receiver operating characteristic (ROC) curve construction. Kaplan–Meier (K–M) and Cox proportional-hazards analyses were performed for prognosis factors. The bioinformatics approach was used to identify targeting miRNA for EPB41L4A-AS1 and UNC5B-AS1. Kaplan–Meier survival curves and Whitney Mann U tests were conducted for validating the statistical significance. In cell culture experiments, the influence of EPB41L4A-AS1 and UNC5B-AS1 on proliferation, migration, and invasion of the osteosarcoma cell line was examined by CCK-8 and Transwell assays.

**Results:**

Levels of EPB41L4A-AS1 and UNC5B-AS1 were upregulated in osteosarcoma patients and cells compared with the healthy participants and normal cell lines. EPB41L4A-AS1 and UNC5B-AS1 have a potent ability to distinguish the patients with osteosarcoma from the health. EPB41L4A-AS1 and UNC5B-AS1 levels correlated with SSS stage. Patients with high levels of EPB41L4A-AS1 and UNC5B-AS1 had significantly shorter survival times. EPB41L4A-AS1 and UNC5B-AS1 were independent prognostic indexes for overall survival. miR-1306-5p was a common target for EPB41L4A-AS1 and UNC5B-AS1. A propulsive impact on cell proliferation, migration, and invasion by EPB41L4A-AS1 and UNC5B-AS1 was observed, but can be rescued by miR-1306-5p.

**Conclusions:**

It was concluded that upregulations of EPB41L4A-AS1 and UNC5B-AS1 expression were diagnostic and prognostic biomarkers for human osteosarcoma. EPB41L4A-AS1 and UNC5B-AS1 contribute to the biological behavior of osteosarcoma via miR-1306-5p.

## Background

Osteosarcoma is the most common malignant tumor of direct origin in bone tissue, presenting mixed osteoblastic/osteolytic lesions [1–3]. The disease is relatively rare, with an incidence of 3 in a million [4]. However, it mainly affects children and adolescents, and also focuses on the elderly [4]. A common site of OS formation is usually near the growth plate of long bones such as the tibia, femur, and humerus [5]. The 5-year relative survival rate for childhood is currently about 67% [6]. Unfortunately, the survival rate of patients with metastatic osteosarcoma has not improved significantly over the past few decades [7, 8]. The discovery of more predictive gene markers can better predict the survival rate of patients with osteosarcoma, which has an important role in the stratified treatment of osteosarcoma.

Prognosis is an assessment or prediction of the possible course of the disease and the chance of recovery or survival from the disease. Both upregulation and downregulation in molecules in cancer patients that associate with tumor grade could serve as prognostic markers [9, 10]. Long non-coding RNA (lncRNA) is a class of RNA molecules with a length greater than 200 nt, a wide range of biological sources, and highly conserved secondary and tertiary structures [11]. LncRNAs have been found to play key roles in a variety of cells in bone tissue. Studies have shown that many lncRNAs are involved in the normal development or balance of the skeletal system, regulate the differentiation of osteoblasts, and participate in the occurrence of osteosarcoma [12]. These lncRNAs play important roles in regulating cell epigenetics, maintaining stem cell pluripotency, regulating cell differentiation, determining cell or tissue fate, and regulating bone remodeling [13]. For instance, lncRNA MEG3 involves in the osteogenic differentiation of mesenchymal stem cells and modulates osteogenic differentiation via ceRNA mechanisms [14]. Its dysregulation has been proposed as a prognostic biomarker for patients with osteosarcoma [15]. LncRNA EPB41L4A-AS1 is a p53 and peroxisome proliferator-activated receptor gamma-coactivator 1-alpha (PGC-1alpha)-inducible gene whose dysregulation is frequently found in many human cancers and is associated with prognosis [16–18]. UNC5B-AS1 is a carcinogen in many cancers, including hepatocellular carcinoma, prostate cancer, and ovarian cancer [19–21]. EPB41L4A-AS1 and UNC5B-AS1 have been reported with dysregulated expression in patients with osteosarcoma [22]. Their clinical roles in osteosarcoma haven’t been uncovered.

In this study, we would demonstrate the expression profiles, the diagnostic and prognostic significance of EPB41L4A-AS1 and UNC5B-AS1 in osteosarcoma, along with their influence on cell aggressive biological behavior in osteosarcoma in humans.

## Methods

### Human osteosarcoma tissue samples

Patients were selected from the Shanghai Baoshan Luodian Hospital. We included the eligible patients who underwent surgery between 2012 and 2014 and fulfilled the following criteria: primary osteosarcoma, no other cancers or major diseases, and no prior therapy. All ages and both localized and metastatic osteosarcoma were included. We identified 113 patients who met these inclusion criteria. Tissue specimens were immediately sectioned, snap frozen and stored at − 80 °C. Informed written consent of each patient and/or their legal guardians was obtained. This study was approved by the ethics and protocol review committee of Shanghai Baoshan Luodian Hospital. Related data were extracted from the document or digital archives at the institutional database. The clinical and tumor characteristics of all patients were recorded and presented in Table 1.

**Table 1:** Correlation between EPB41L4A-AS1 or UNC5B-AS1 expression levels and clinical features in patients with osteosarcoma

Parameter	N	Low EPB41L4A-AS1 (n = 52)	High EPB41L4A-AS1 (n = 61)	*P*	Low UNC5B-AS1 (n = 49)	High UNC5B-AS1 (n = 64)	*P*
*Age (years)*				0.840			0.062
< 18	62	28	34		22	40	
≥ 18	51	24	27		27	24	
*Gender*				0.245			0.555
Male	52	27	35		21	31	
Female	61	25	36		28	33	
*Tumor site*				0.345			0.964
Femur	51	22	29		22	29	
Tibia	23	8	15		11	12	
Humeral bone	12	6	6		5	7	
other	27	16	11		11	16	
*SSS stage*				0.048*			0.013*
I–II	81	42	39		41	40	
III	32	10	22		8	24	
*LDH*				0.749			0.051
< 850 IU/L	67	30	37		24	43	
≥ 850 IU/L	46	22	24		25	21	
*ALP*				0.695			0.699
< 280 IU/L	76	34	42		32	44	
≥ 280 IU/L	37	18	19		17	20	

LDH, lactate dehydrogenase; ALP, alkaline phosphatase

*P < 0.05. SSS: surgical staging system

### Human osteosarcoma cell lines

Human osteoblast cell line hFOB1.19 and five kinds of human osteosarcoma cell lines, MG-63, Saos-2, MNNG/HOS, U-2 OS, and 143B were purchased from the Chinese National Collection of Authenticated Cell Cultures (Beijing, China).hFOB1.19 was cultured in DMEM/F12 (#12400024, GIBCO, USA), supplemented with 0.3 mg/ml G418 and 10% FBS, in a humidified 5% CO_2_/air atmosphere at 33.5 °C. MG-63 and MNNG/HOS were cultured in MEM (#41500034, GIBCO, USA), supplemented with 10% FBS, in a humidified 5% CO_2_/air atmosphere at 37 °C. Saos-2 and U-2 OS were cultured in McCoy's 5a Medium (12330-031, Invitrogen, USA) supplement with 15% FBS and 10% FBS, respectively. 143B was cultured in McCoy's 5a Medium (12330-031, Invitrogen, USA) supplement with 10% FBS (Gibco).

### Cell transfection

EPB41L4A-AS1 and UNC5B-AS1 were silenced using specific siRNA pools, si-EPB41L4A-AS1 (5′-GGATGTCCTTGGTGAGGATTT-3′) and si-UNC5B-AS1 (5′-GAUCCUGCCUCAGGGAAAU-3′), or equal amounts of nonspecific sham siRNA (si-ctr, 5′-TTCTCCGAACGTGTCACGT-3′) obtained from Ibsbio (Shanghai, China), along with miR-1306-3p inhibitor (in-miR-1306-3p, 5′-CACCACCAGAGCCAACGU-3′) and inhibitor negative control (in-ctr, 5′-UCACAACCUCCUAGAAAGAGUAGA -3′). MG-63 and 143B cells were transfected with 50 nM (final concentration) of siRNAs or inhibitors using Lipofectamine™ RNAiMAX (Invitrogen) and respective medium. Cells were cultured for 48 h before to confirm the efficiency by quantitative reverse-transcriptase polymerase chain reaction (qRT-PCR).

### RNA isolation and qRT-PCR

Total RNAs of cells and tissues were extracted using TRIzol RNA Isolation Reagents (Thermo Fisher Scientific, USA). The RNA is converted to cDNA using GoScript reverse transcriptase (Promega, USA). The expression of each of the individual transcripts was quantified by qRT-PCR (Applied Biosystems 7500 Real-Time) using the primers and GoTaq qPCR master mix (Promega, USA). Human GAPDH mRNA was used as an internal control to normalize UNC5B-AS1 levels, ACTB mRNA to normalize EPB41L4A-AS1 levels, and U6 mRNA to normalize miR-1306-3p levels. Primers were: 5′-CCACCCTGAGTCTGGTGAGT-3′ (forward for EPB41L4A-AS1) and 5′-CCTGGCATAGTCGATGATGTA-3′ (reverse for EPB41L4A-AS1); 5′-GAAGGTGAAGGTCGGAGTC-3′ (forward for GAPDH) and 5′-GAAGATGGTGA TGGGATTTC-3′ (reverse for GAPDH); 5′-GATCCTGCCTCAGGGAAA-3′ (forward for UNC5B-AS1) and 5′-GCTCAAGAGGTTGGGACT-3' (reverse for UNC5B-AS1); 5'-CGTCGACAACGGCTCCGGCATG-3′ (forward for ACTB) and 5'-GGGCCTCGTCACCCACATAGGAG-3′ (reverse for ACTB); 5'-CGCGGTGGTGGTCTCG-3′ (forward for miR-1306-3p) and 5′-AGTGCAGGGTCCGAGGTATT-3′ (reverse for miR-1306-3p); 5′-CAAGGATGACACGCAAA-3′ (forward for U6) and 5'-TCAACTGGTGTCGTGG-3' (reverse for U6). The data were presented as results after 2^−ΔΔCT^.

### Bioinformatics

To predict target miRNAs for EPB41L4A-AS1 and UNC5B-AS1, an independent bioinformatic lncRNA prediction tool, lncRNASNP2 (http://bioinfo.life.hust.edu.cn/lncRNASNP#!/), was applied.

### Biotinylated-probe pull-down assay

Biotin-labeled miR-1306-5p was transfected into MG-63 and 143B cells. The cell lysates were incubated with streptavidin-coupled magnetic beads (Thermo Fisher Scientific, USA) for an additional 16 h at 4 °C with rotation. The bead-RNA complexes were harvested, washed, DNase treated, and purified. The expression levels of EPB41L4A-AS1 and UNC5B-AS1 were detected by qRT-PCR.

### Proliferation assay

The MG-63 and 143B cells, after being transfected and transferred into a 96-well plate (2 × 10^3^ cells/well) for 24 h, were incubated with 10 μL of CCK-8 kit (#Ab228554, Abcam, Cambridge, USA) for 3 h. Then, the absorbance increase was measured at 460 nm. Likewise, the absorbance increases of the cells at 48 h and 72 h after the inoculation were monitored. Finally, the absorbance values, directly proportional to the number of living cells, were used to construct the proliferation curves of the cells.

### Migration assay

Invasion assays with MG-63 and 143B cells were carried out using the Corning Transwell Permeable Support (Cat 3422, Corning, USA). In brief, the inserts were washed with PBS and the chambers were equilibrated with 0.5 mL of the serum-free medium at 37 °C for 2 h. After starvation with serum-free DMEM for 4 h, subconfluent cells were trypsinized and resuspended in 0.1% FBS media. 0.5 mL of 10% FBS-containing media was added to the lower chambers. 3 × 10^4^ cells in 0.5 mL of serum-free media were added to the upper chamber. The chambers were incubated for 24 h at 37 °C, 5% CO_2_. Non-migrated cells were removed from the upper chamber, then the migrated cells were fixed and stained. The counting of cells was done randomly in five microscopic fields.

### Invasion assay

Invasion assays with MG-63 and 143B cells were carried out using the Corning® BioCoat™ Matrigel® Invasion Chamber (Bedford, USA). The Matrigel inserts were rehydrated. 3 × 10^4^ cells in 0.5 mL of serum-free media were added to the upper chamber. The chemoattractant (15% FBS) was added to the wells of the Falcon TC Companion Plate. The whole chamber was incubated for 24 h at 37 °C, 5% CO_2_ atmosphere. After the removal of non-invading cells and staining of the invading cells, direct counting of the cells was performed under the microscope.

### Statistical analyses

Data are presented as mean ± standard deviation in tables and figures. Demographics, drinking history markers, and clinical differences were analyzed using one-way ANOVA with Bonferroni's correction. EPB41L4A-AS1 and UNC5B-AS1 levels among different groups were calculated using paired t-tests. EPB41L4A-AS1 and UNC5B-AS1 levels were correlated with clinical markers using the Chi-square test. Receiver-operating characteristic (ROC) curves were constructed in the single or combined form for EPB41L4A-AS1 and UNC5B-AS1 to examine the sensitivity and specificity of the two lncRNAs as diagnostic biomarkers. Kaplan–Meier survival curves were further constructed and compared using the log-rank tests. Multivariate Cox proportional hazards analysis was used to determine which variables were significant in predicting the prognosis of osteosarcoma. Significance was set at *P* < 0.05.

## Results

### Upregulated expression levels of EPB41L4A-AS1 and UNC5B-AS1 in human osteosarcoma

**Fig. 1:**
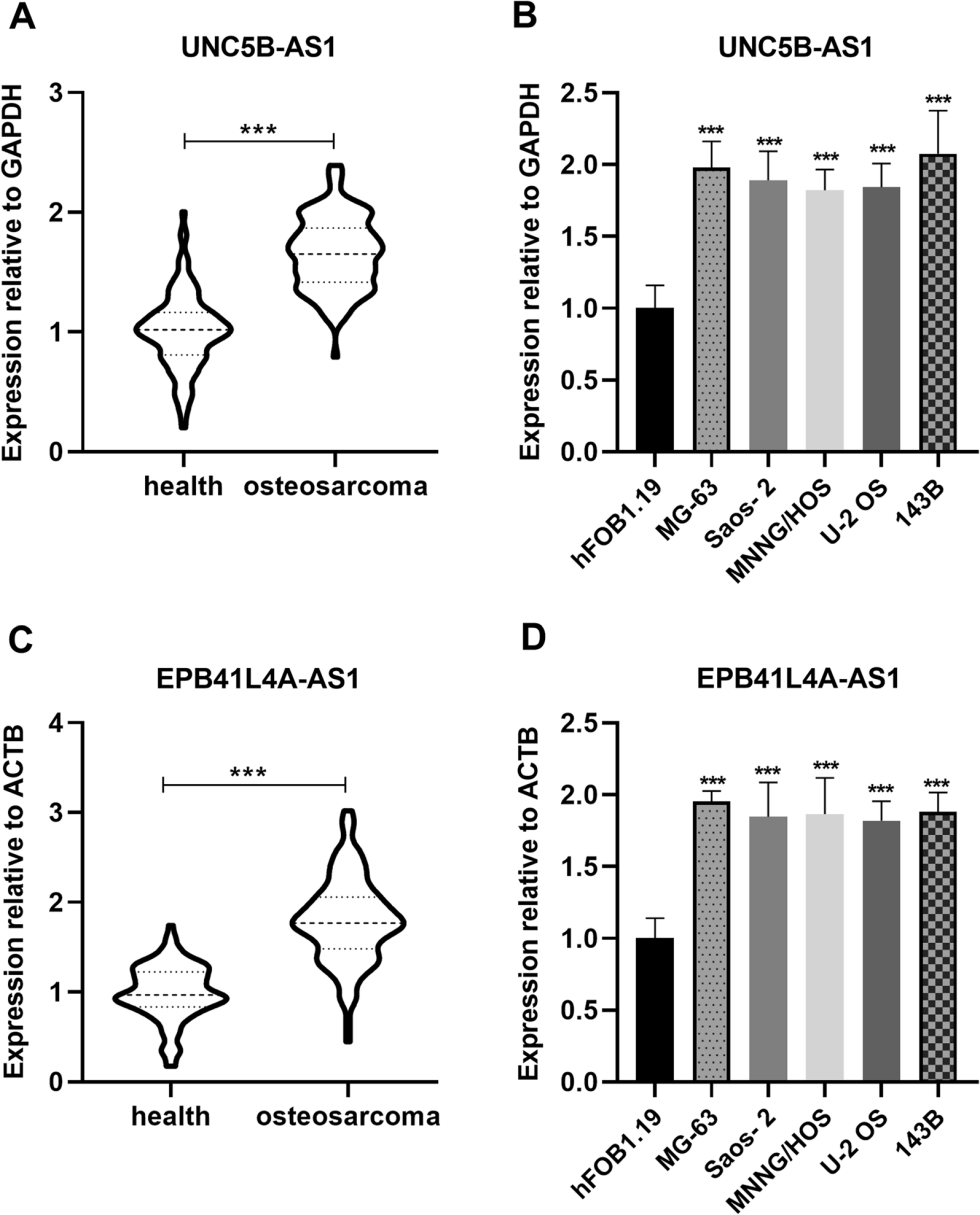
EPB41L4A‐AS1 and UNC5B-AS1 were highly expressed in human osteosarcoma. **A** EPB41L4A‐AS1 expression levels were determined by qRT-PCR in osteosarcoma tumor samples and normal tissues. **B** EPB41L4A‐AS1 expression levels determined by qRT-PCR in osteosarcoma cell lines (MG-63, Saos-2, MNNG/HOS, U-2 OS, and 143B) and human osteoblast cell line hFOB1.19. **C** UNC5B-AS1 expression levels were determined by qRT-PCR in osteosarcoma tumor samples and normal tissues. **D** UNC5B-AS1 expression levels were determined by qRT-PCR in osteosarcoma cell lines (MG-63, Saos-2, MNNG/HOS, U-2 OS, and 143B) and human osteoblast cell line hFOB1.19. ***P < 0.001

qRT-PCR was used to detect the expression levels of EPB41L4A-AS1 and UNC5B-AS1 in tissues and cells. The analysis revealed that EPB41L4A-AS1 levels were highly expressed in human osteosarcoma tissues and cells when compared with the corresponding normal ones (Fig. 1A, B). For UNC5B-AS1, increased expression levels were also found in human osteosarcoma tissues and cells when compared with the corresponding normal ones (Fig. 1C, D). Therefore, EPB41L4A-AS1 and UNC5B-AS1 both show upregulated expression levels in human osteosarcoma.

### Diagnostic and prognostic significance of EPB41L4A-AS1 and UNC5B-AS1 in human osteosarcoma

**Fig. 2:**
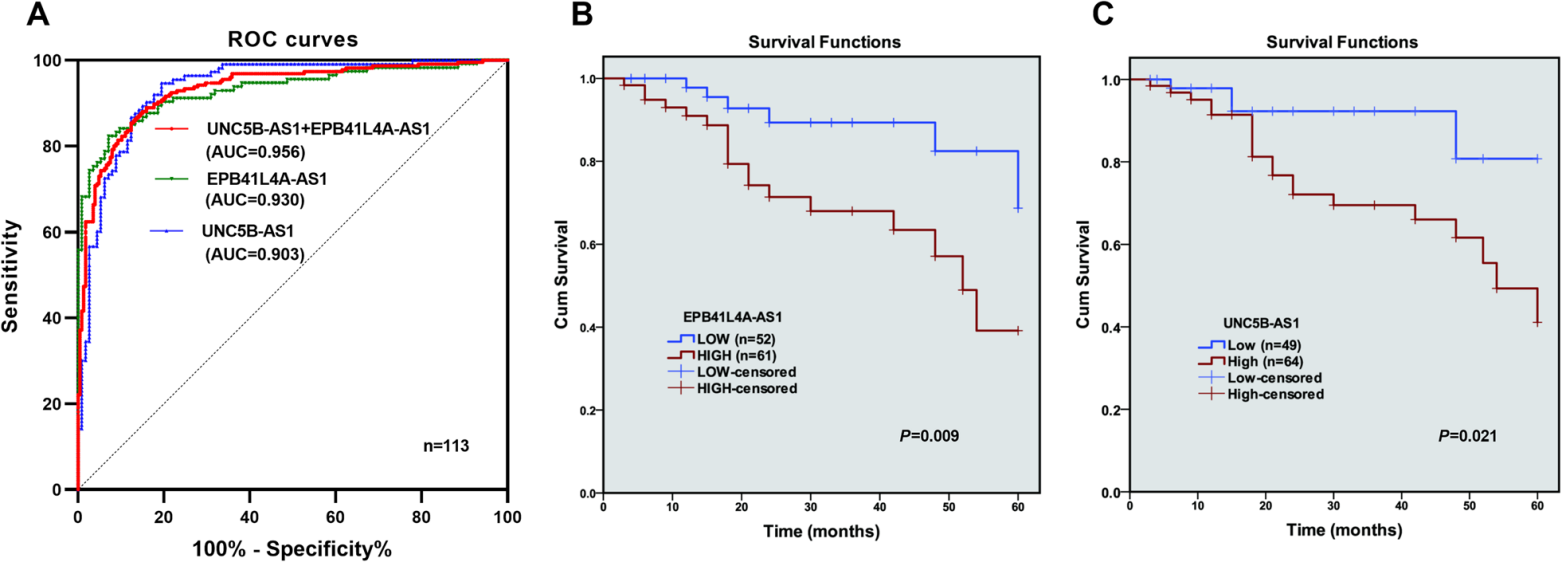
The diagnostic and prognostic value of EPB41L4A‐AS1 and UNC5B-AS1 for human osteosarcoma. **A** ROC curves were constructed based on the expression of EPB41L4A‐AS1 and UNC5B-AS1. **B** Kaplan–Meier analysis of overall survival in osteosarcoma patients based on EPB41L4A‐AS1 expression. P-value was 0.009. **C** Kaplan–Meier analysis of overall survival in osteosarcoma patients based on UNC5B-AS1 expression. *P*-value was 0.021

The dysregulations of EPB41L4A-AS1 and UNC5B-AS1 implied their potential significance in human osteosarcoma. To confirm the diagnostic potential, the application of ROC for EPB41L4A-AS1 revealed that the AUC was 0.930 (95% CI 0.896–0.964; *P* < 0.001, Fig. 2A) with a sensitivity of 82.30% and specificity of 92.92%. For UNC5B-AS1, the AUC was 0.935 (95% CI 0.903–0.967; *P* < 0.001, Fig. 2A) with a sensitivity of 94.69% and specificity of 80.53%. When EPB41L4A-AS1 and UNC5B-AS1 are combined, the AUC was 0.932 (95% CI 0.909–0.956; *P* < 0.001, Fig. 2A) with a sensitivity of 86.73% and specificity of 86.28%. At a 5-year follow-up, a total of 24 (21.2%) deaths were observed. When patients were categorized into groups based on the median values of EPB41L4A-AS1 and UNC5B-AS1 expression levels, EPB41L4A-AS1 (*P* = 0.048) and UNC5B-AS1 (*P* = 0.013) were found to be significantly associated with surgical staging system (SSS) stage (Table 1). Further, the multi-univariate analysis identified EPB41L4A-AS1 and UNC5B-AS1 expression levels were related to overall survival (Table 2): EPB41L4A-AS1 (HR = 5.997, 95% CI 1.845–19.486, *P* = 0.003) and UNC5B-AS1 (HR = 5.911, 95% CI 1.828–19.181, *P* = 0.003). The Kaplan–Meier survival curves for these two lncRNAs are shown in Fig. 2B (*P* = 0.009 for EPB41L4A-AS1) and Fig. 2C (*P* = 0.021 for UNC5B-AS1). These results indicate that both EPB41L4A-AS1 and UNC5B-AS1 have demonstrated high discriminatory ability for osteosarcoma and can predict poor prognosis.

**Table 2:** Multivariate Cox analysis of prognostic factors in patients with osteosarcoma based on the overall survival

Parameter	Cumulative overall survival		
	HR	95%CI	*P*
EPB41L4A-AS1 (High vs. Low)	5.997	1.845–19.486	0.003**
UNC5B-AS1 (High vs. Low)	5.911	1.828–19.18	0.003**
Age (≥ 18 years vs. < 18 years)	1.144	0.437–2.996	0.784
Gender (Male vs. Female)	2.085	0.761–5.713	0.153
*Tumor site*			
Femur	Reference		
Tibia	0.509	0.134–1.927	0.320
Humeral bone	1.078	0.265–4.381	0.917
Other	1.134	0.226–5.684	0.878
SSS stage (III vs. I–II)	4.676	1.430–15.291	0.011*
LDH (≥ 850 IU/L vs. < 850 IU/L)	1.842	0.667–5.086	0.238
ALP (≥ 280 IU/L vs. < 280 IU/L)	1.452	0.566–3.724	0.437

LDH, lactate dehydrogenase; ALP, alkaline phosphatase

*P < 0.05, **P < 0.01. SSS: surgical staging system

### Common target miRNA of EPB41L4A-AS1 and UNC5B-AS1

**Fig. 3:**
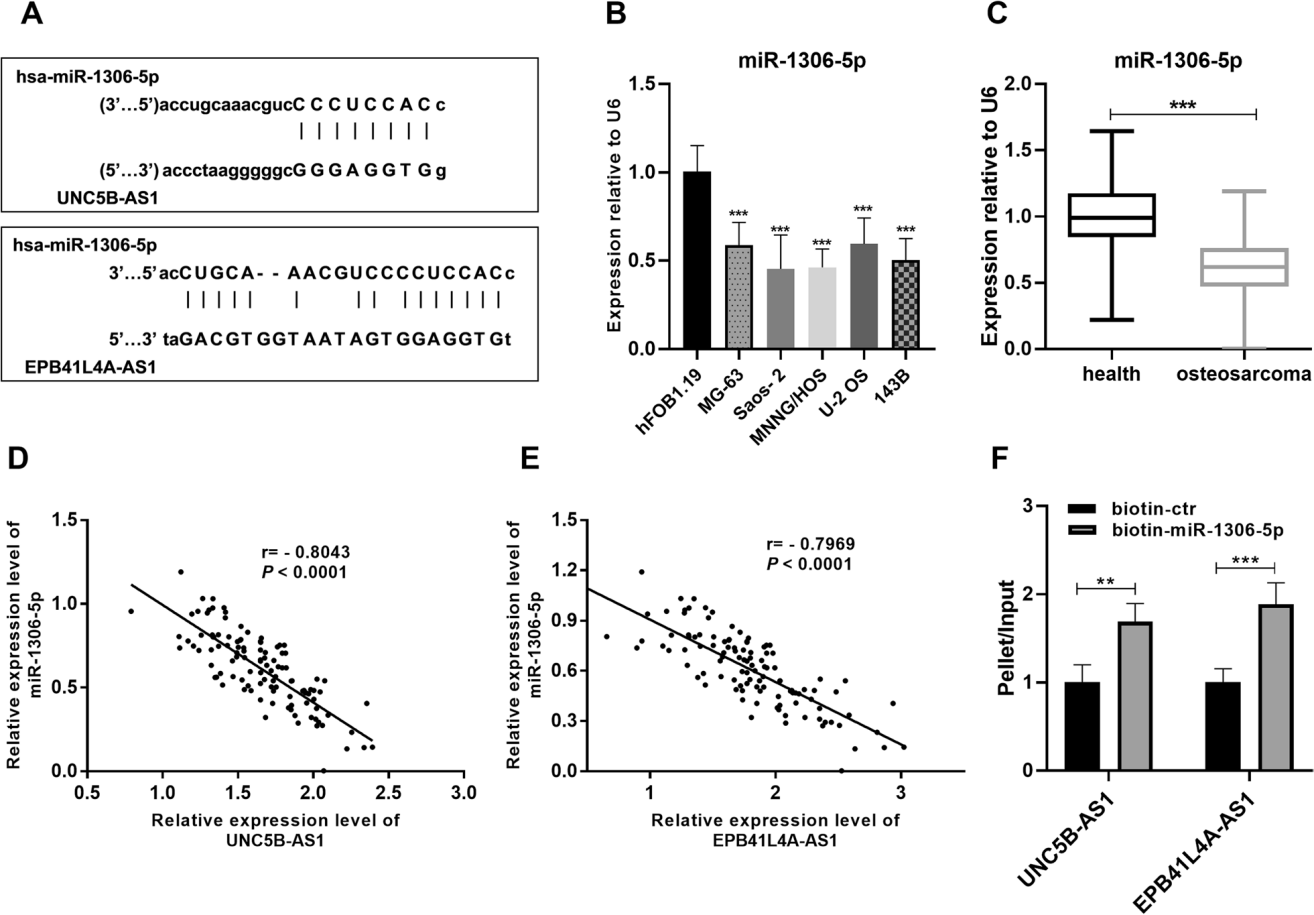
MiR-1306-5p was a target for EPB41L4A‐AS1 and UNC5B-AS1. **A** The binding sites for miR-1306-5p with EPB41L4A‐AS1 and UNC5B-AS1 were predicted by lncRNASNP2. **B** MiR-1306-5p expression levels determined by qRT-PCR in osteosarcoma cell lines (MG-63, Saos-2, MNNG/HOS, U-2 OS, and 143B) and human osteoblast cell line hFOB1.19. **C** MiR-1306-5p expression levels were determined by qRT-PCR in osteosarcoma tumor samples and normal tissues. **D** The expression levels of miR-1306-5p and EPB41L4A‐AS1 were negatively correlated. **E** The expression levels of miR-1306-5p and UNC5B-AS1 were negatively correlated. **F** Pulldown assay in MG-63 cells

Given the theory that lncRNAs harbor microRNA (miRNA)-response elements, lncRNAs can act as competitive endogenous RNAs (ceRNA) to compete for a common pool of miRNAs. After the analysis of lncRNASNP2, miR-1306-5p was found to be the common target miRNA of EPB41L4A-AS1 and UNC5B-AS1 (Fig. 3A). The expression of miR-1306-5p was upregulated in human osteosarcoma cells (Fig. 3B) and tissues (Fig. 3C) compared to the corresponding normal ones. The level of miR-1306-5p was negatively correlated with EPB41L4A-AS1 level (Fig. 3D) and UNC5B-AS1 level (Fig. 3E). The pulldown of MG-63 cells revealed that EPB41L4A-AS1 and UNC5B-AS1 were specifically co-precipitated with the biotinylated-tagged miR-1306-5p (Fig. 3F).

### EPB41L4A-AS1 promotes cancer properties of osteosarcoma cells by binding miR-1306-5p

**Fig. 4:**
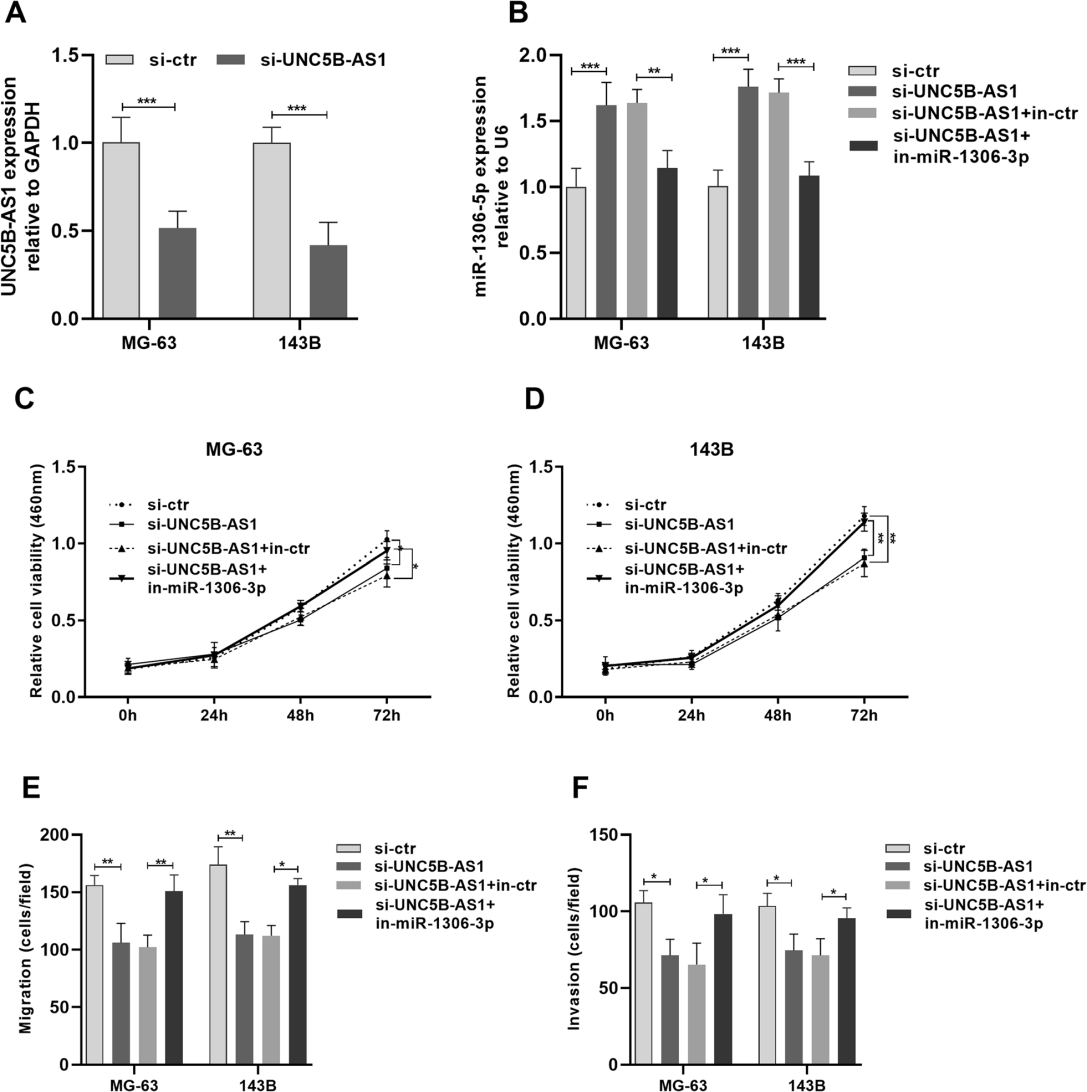
EPB41L4A‐AS1 accelerated osteosarcoma progression via targeting miR-1306-5p. **A** The transfection efficiency of EPB41L4A‐AS1 and miR-1306-5p was verified by qRT-PCR. **B**, **C** CCK-8 assays were carried out to assess the proliferative capacity of MG-63 and 143B cells. **D** Cell migration was detected by the transwell chamber assays. **E** Cell invasion was detected by transwell chamber assays. * P < 0.01, **P < 0.01, ***P < 0.001

We further knocked down EPB41L4A-AS1 and miR-1306-5p in MG63 and 143B cells to explore the association between EPB41L4A-AS1 and miR-1306-5p (Fig. 4A, B). CCK8 assay revealed that EPB41L4A-AS1 inhibition hindered the proliferation of cells, whereas miR-1306-5p knockdown partially impaired this phenomenon (Fig. 4C, D). An inhibitory effect of downregulating EPB41L4A-AS1 on cell migration was found, whereas expression miR-1306-5p knockdown substantially reversed this inhibitory effect (Fig. 4E), consistent with cell invasion assay results (Fig. 4F). Hence, EPB41L4A-AS1 promotes the proliferation, migration, and invasion of osteosarcoma cells partly via miR-1306-5p.

### UNC5B-AS1 promotes cancer properties of osteosarcoma cells by binding miR-1306-5p

**Fig. 5:**
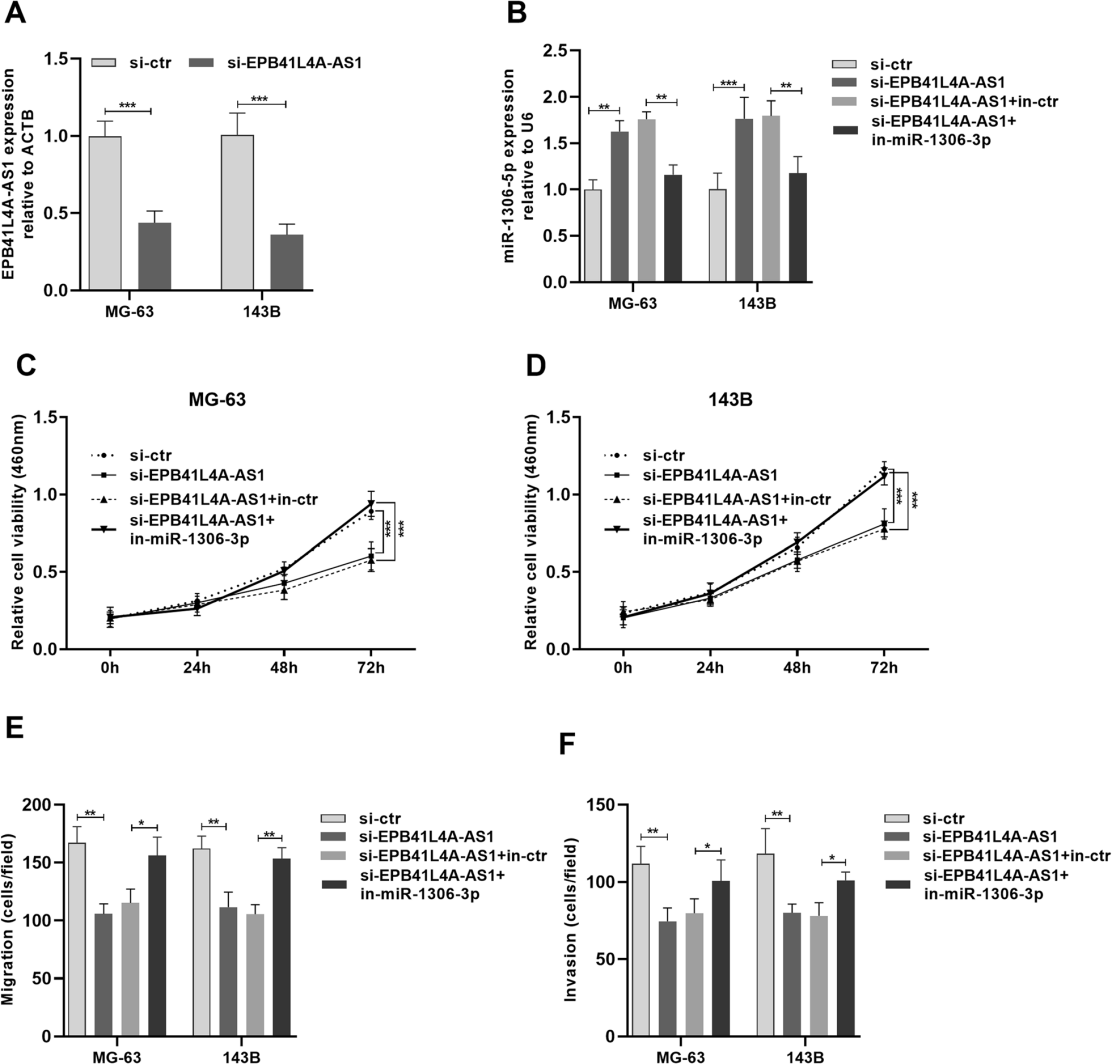
UNC5B-AS1 accelerated osteosarcoma progression via targeting miR-1306-5p. **A** The transfection efficiency of UNC5B-AS1 and miR-1306-5p was verified by qRT-PCR. **B**, **C** CCK-8 assays were carried out to assess the proliferative capacity of MG-63 and 143B cells. **D** Cell migration was detected by the transwell chamber assays. **E** Cell invasion was detected by transwell chamber assays. * P < 0.01, **P < 0.01, ***P < 0.001

UNC5B-AS1 and miR-1306-5p were knocked down in MG63 and 143B cells to explore the association between UNC5B-AS1 and miR-1306-5p (Fig. 5A, B). CCK8 assay revealed that UNC5B-AS1 inhibition hindered the proliferation of cells, whereas miR-1306-5p knockdown partially impaired this phenomenon (Fig. 5C, D). An inhibitory effect of downregulating UNC5B-AS1 on cell migration was found, whereas expression miR-1306-5p knockdown substantially reversed this inhibitory effect (Fig. 5E), consistent with cell invasion assay results (Fig. 5F). Therefore, UNC5B-AS1 promotes proliferation, migration, and invasion of osteosarcoma cells partly via miR-1306-5p.

## Discussion

Previous studies have reported that lncRNAs participate in the regulation of transcription and translation of protein-coding genes in mammalian genomes. LncRNAs regulate protein coding genes at the transcriptional or post-transcriptional level, and then participate in multiple biological processes, including cell differentiation, development, and human disease [23, 24]. Human genome studies have shown that approximately 18% of protein-coding genes that produce lncRNAs (10/57) are associated with cancer, while only 9% of all human protein-coding genes are associated with cancer, [25]. This demonstrates that genes implicated in cancer development have a greater tendency to produce lncRNAs. LncRNAs are involved in osteosarcoma development, metastasis, invasion, apoptosis, tumor size, and proliferation [26]. LncRNAs have great potential to be considered biomarkers because they are more stable and highly tissue/cell-specific. In The role of lncRNAs as prognostic biomarkers in osteosarcoma treatment. In this study, the expression levels of EPB41L4A-AS1 and UNC5B-AS1 in osteosarcoma were elevated. Further investigation of this dysregulated expression revealed that EPB41L4A-AS1 and UNC5B-AS1 have the potential as diagnostic and prognostic factors. Through cell function studies, it was found that EPB41L4A-AS1 and UNC5B-AS1 can promote the proliferation, migration, and invasion of human osteosarcoma cells by binding to miR-1306-5p.

EPB41L4A-AS1 has been reported as hematopoiesis/oncology-related lncRNA and it was significantly upregulated in myelodysplastic syndrome [27]. In this study, EPB41L4A-AS1 was upregulated in osteosarcoma, consistent with that in colorectal cancer [16]. A report by Shao et al. supported the upregulation of EPB41L4A-AS1 in osteosarcoma [22]. This dysregulation suggests its clinical potential. ROC curve analysis showed that EPB41L4A-AS1 expression level gives good performance in distinguishing patients with osteosarcoma from healthy participants. Based on the median value of EPB41L4A-AS1 expression level, the 113 patients were classified to be the high EPB41L4A-AS1 group and the low EPB41L4A-AS1 group. EPB41L4A-AS1 was significantly correlated with the SSS stage. The survival analysis by the Kaplan–Meier method and multi-univariate Cox proportional hazards regression analysis revealed that EPB41L4A-AS1 was an independent prognostic factor for osteosarcoma. It is reported that EPB41L4A-AS1 can enhance the proliferation of bone marrow-derived mesenchymal stem cells [28]. Our results also revealed the promoting role of EPB41L4A-AS1 in the proliferation of osteosarcoma cells. Besides, we found that EPB41L4A-AS1 can promote the migration and invasion of osteosarcoma cells. Therefore, our study revealed that EPB41L4A-AS1 can promote the progression of osteosarcoma, and serve as a diagnostic and prognostic biomarker.

In cancer research, UNC5B-AS1 usually appears as a carcinogen. For example, it accelerates the progression of hepatocellular carcinoma [19], papillary thyroid cancer [29], and lung cancer [30]. In osteosarcoma, UNC5B-AS1 is expressed highly from the results of tissues and cells by qRT-PCR. Our further investigation has shown that UNC5B-AS1 has a distinct discriminative effect on patients with osteosarcoma. Moreover, high expression of UNC5B-AS1 is associated with a high SSS stage and poor prognosis in patients with osteosarcoma. UNC5B-AS1 appeared as a powerful prognostic factor in multivariate COX analysis of osteosarcoma prognosis-related factors. The prognostic value of UNC5B-AS1 has also been reported by Shao et al. in a panel of lncRNAs [22]. The results of cellular functional studies suggest that UNC5B-AS1 can promote the growth and metastasis of osteosarcoma cells. Hence, UNC5B-AS1 acts as a carcinogen in osteosarcoma.

The ceRNA action of lncRNAs occurs from lncRNAs that share miRNA response elements with coding RNAs [31]. These lncRNAs contain similar miRNA target sequences and act as “sponges” for miRNAs, which then prevent miRNAs from acting on mRNAs [32]. UNC5B-AS1 was sponges for miR-4455 in cervical cancer [33], miR-218-5 in lung cancer [30], and miR-4306 in hepatocellular carcinoma [19]. EPB41L4A‐AS1 targeted miR‐146a and regulated the proliferation of bone marrow-derived mesenchymal stem cells [28]. Interestingly, our study found that miR-1306-5p was the target of both UNC5B-AS1 and EPB41L4A‐AS1. The cell experiment revealed that miR-1306-5p can recover the inhibitory effect of UNC5B-AS1 and EPB41L4A‐AS1 on cell proliferation, migration, and invasion. Taken together, these results suggest that UNC5B-AS1 and EPB41L4A‐AS1 may play the promoting role in osteosarcoma via miR-1306-5p. However, limitations of the present study, including absence of in vivo experiments and a large number of multi-agency samples, should be considered. These will be the focus of future research.

## Conclusions

The expression levels of UNC5B-AS1 and EPB41L4A‐AS1 were determined, and both showed upregulated expression in human osteosarcoma. Further, the UNC5B-AS1 and EPB41L4A‐AS1 diagnostic and prognostic significance was confirmed in human osteosarcoma. UNC5B-AS1 and EPB41L4A‐AS1 contributed to the progression of osteosarcoma via miR-1306-5p. This study represents novel therapeutic targets, which are critical for developing novel strategies for the diagnosis, prognosis, and treatment of human osteosarcoma.

## Data Availability

The datasets used and/or analysed during the current study are available from the corresponding author on reasonable request.

## Abbreviations

ceRNA: Competitive endogenous RNAs
in-ctr: Inhibitor negative control
in-miR-1306-3p: MiR-1306-3p inhibitor
K–M: Kaplan–Meier
lncRNA: Long non-coding RNA
miRNA: MicroRNA
PGC-1alpha: Proliferator-activated receptor gamma-coactivator 1-alpha
ROC: Receiver operating characteristic

## References

[1] Pin F, Prideaux M, Bonewald LF, Bonetto A (2021). Osteocytes and cancer. Curr Osteoporos Rep.

[2] Eaton BR, Schwarz R, Vatner R, Yeh B, Claude L, Indelicato DJ (2021). Osteosarcoma. Pediatric Blood Cancer.

[3] Belayneh R, Fourman MS, Bhogal S, Weiss KR (2021). Update on osteosarcoma. Curr Oncol Rep.

[4] Sadykova LR, Ntekim AI, Muyangwa-Semenova M, Rutland CS, Jeyapalan JN, Blatt N (2020). Epidemiology and risk factors of osteosarcoma. Cancer Invest.

[5] Kansara M, Teng MW, Smyth MJ, Thomas DM (2014). Translational biology of osteosarcoma. Nat Rev Cancer.

[6] Siegel RL, Miller KD, Fuchs HE, Jemal A (2021). Cancer statistics, 2021. CA Cancer J Clin.

[7] Song K, Song J, Lin K, Chen F, Ma X, Jiang J (2019). Survival analysis of patients with metastatic osteosarcoma: a surveillance, epidemiology, and end results population-based study. Int Orthop.

[8] Smeland S, Bielack SS, Whelan J, Bernstein M, Hogendoorn P, Krailo MD (2019). Survival and prognosis with osteosarcoma: outcomes in more than 2000 patients in the EURAMOS-1 (European and American Osteosarcoma Study) cohort. Eur J Cancer (Oxford, England: 1990).

[9] Yang G, Wu Y, Wan R, Sang H, Liu H, Huang W (2021). The role of non-coding RNAs in the regulation, diagnosis, prognosis and treatment of osteosarcoma. Int J Oncol.

[10] Asnafi AA, Behzad MM, Ghanavat M, Shahjahani M, Saki N (2019). Singe nucleotide polymorphisms in osteosarcoma: pathogenic effect and prognostic significance. Exp Mol Pathol.

[11] Dykes IM, Emanueli C (2017). Transcriptional and post-transcriptional gene regulation by long non-coding RNA. Genom Proteom Bioinform.

[12] Moonmuang S, Chaiyawat P, Jantrapirom S, Pruksakorn D, Lo PL (2021). Circulating long non-coding RNAs as novel potential biomarkers for osteogenic sarcoma. Cancers.

[13] Aurilia C, Donati S, Palmini G, Miglietta F, Iantomasi T, Brandi ML (2021). The involvement of long non-coding RNAs in bone. Int J Mol Sci.

[14] Sun H, Peng G, Wu H, Liu M, Mao G, Ning X (2020). Long non-coding RNA MEG3 is involved in osteogenic differentiation and bone diseases. Biomed Rep.

[15] Jiang M, Wang YR, Xu N, Zhou L, An Q (2019). Long noncoding RNA MEG3 play an important role in osteosarcoma development through sponging microRNAs. J Cell Biochem.

[16] Bin J, Nie S, Tang Z, Kang A, Fu Z, Hu Y (2021). Long noncoding RNA EPB41L4A-AS1 functions as an oncogene by regulating the Rho/ROCK pathway in colorectal cancer. J Cell Physiol.

[17] Deva Magendhra Rao AK, Patel K, Korivi Jyothiraj S, Meenakumari B, Sundersingh S, Sridevi V (2019). Identification of lncRNAs associated with early-stage breast cancer and their prognostic implications. Mol Oncol.

[18] Yang F, Lv S (2022). LncRNA EPB41L4A-AS1 regulates cell proliferation, apoptosis and metastasis in breast cancer. Ann Clin Lab Sci.

[19] Huang X, Pan J, Wang G, Huang T, Li C, Wang Y (2021). UNC5B-AS1 promotes the proliferation, migration and EMT of hepatocellular carcinoma cells via regulating miR-4306/KDM2A axis. Cell Cycle (Georgetown, Tex).

[20] Tan SF, Ni JX, Xiong H (2020). LncRNA UNC5B-AS1 promotes malignant progression of prostate cancer by competitive binding to caspase-9. Eur Rev Med Pharmacol Sci.

[21] Wang H, Su H, Tan Y (2020). UNC5B-AS1 promoted ovarian cancer progression by regulating the H3K27me on NDRG2 via EZH2. Cell Biol Int.

[22] Hong-Bin S, Wan-Jun Y, Chen-Hui D, Xiao-Jie Y, Shen-Song L, Peng Z (2022). Identification of an iron metabolism-related lncRNA signature for predicting osteosarcoma survival and immune landscape. Front Genet.

[23] Diederichs S, Bartsch L, Berkmann JC, Fröse K, Heitmann J, Hoppe C (2016). The dark matter of the cancer genome: aberrations in regulatory elements, untranslated regions, splice sites, non-coding RNA and synonymous mutations. EMBO Mol Med.

[24] Schmitz SU, Grote P, Herrmann BG (2016). Mechanisms of long noncoding RNA function in development and disease. Cell Mol Life Sci.

[25] Khachane AN, Harrison PM (2010). Mining mammalian transcript data for functional long non-coding RNAs. PLoS ONE.

[26] Yang Z, Li X, Yang Y, He Z, Qu X, Zhang Y (2016). Long noncoding RNAs in the progression, metastasis, and prognosis of osteosarcoma. Cell Death Dis.

[27] Szikszai K, Krejcik Z, Klema J, Loudova N, Hrustincova A, Belickova M (2020). LncRNA profiling reveals that the deregulation of H19, WT1-AS, TCL6, and LEF1-AS1 is associated with higher-risk myelodysplastic syndrome. Cancers.

[28] Cui P, Zhao X, Liu J, Chen X, Gao Y, Tao K (2020). miR-146a interacting with lncRNA EPB41L4A-AS1 and lncRNA SNHG7 inhibits proliferation of bone marrow-derived mesenchymal stem cells. J Cell Physiol.

[29] Wang Y, Bhandari A, Niu J, Yang F, Xia E, Yao Z (2019). The lncRNA UNC5B-AS1 promotes proliferation, migration, and invasion in papillary thyroid cancer cell lines. Hum Cell.

[30] Tan JJ, Long SZ, Zhang T (2020). Effects of LncRNA UNC5B-AS1 on adhesion, invasion and migration of lung cancer cells and its mechanism. Chin J Appl Physiol.

[31] Qi X, Zhang DH, Wu N, Xiao JH, Wang X, Ma W (2015). ceRNA in cancer: possible functions and clinical implications. J Med Genet.

[32] Wang JY, Yang Y, Ma Y, Wang F, Xue A, Zhu J (2020). Potential regulatory role of lncRNA-miRNA-mRNA axis in osteosarcoma. Biomed Pharmacother.

[33] Fu J, Zhang Y, Wang M, Hu J, Fang Y (2021). Inhibition of the long non-coding RNA UNC5B-AS1/miR-4455/RSPO4 axis reduces cervical cancer growth in vitro and in vivo. J Gene Med.

